# COVID-19-Associated Subacute Thyroiditis: Evidence-Based Data From a Systematic Review

**DOI:** 10.3389/fendo.2021.707726

**Published:** 2021-09-29

**Authors:** Pierpaolo Trimboli, Carlo Cappelli, Laura Croce, Lorenzo Scappaticcio, Luca Chiovato, Mario Rotondi

**Affiliations:** ^1^ Clinic for Endocrinology and Diabetology, Lugano Regional Hospital, Ente Ospedaliero Cantonale, Lugano, Switzerland; ^2^ Faculty of Biomedical Sciences, Università della Svizzera Italiana (USI), Lugano, Switzerland; ^3^ Department of Clinical and Experimental Sciences, University of Brescia, Brescia, Italy; ^4^ Unit of Internal Medicine and Endocrinology, Laboratory for Endocrine Disruptors, Istituti Clinici Scientifici Maugeri Istituto di Ricovero e Cura a Carattere Scientifico, (IRCCS), Pavia, Italy; ^5^ Department of Internal Medicine and Therapeutics, University of Pavia, Pavia, Italy; ^6^ Division of Endocrinology and Metabolic Diseases, University Hospital “Luigi Vanvitelli”, University of Campania “L. Vanvitelli”, Naples, Italy

**Keywords:** subacute thyroiditis (SAT), subacute thyroiditis de Quervain, SARS-CoV-2, COVID-19, thyroid

## Abstract

Subacute thyroiditis (SAT) is a thyroid disease of viral or post-viral origin. Whether SAT represents a complication of coronavirus disease 2019 (COVID-19) caused by severe acute respiratory syndrome coronavirus 2 (SARS-CoV-2) is still unclear. Our aim was to systematically review the literature to 1) explore the size of the literature about SAT in COVID-19 and 2) evaluate the clinical characteristics of SAT. PubMed/MEDLINE, Embase, and Scopus were searched until April 20, 2021. Original papers, case reports, and case series reporting SAT in COVID-19 patients were included. Authors and their country, journal, year of publication, COVID-19 and SAT clinical presentation, thyroid function, therapy, and follow-up data were extracted. Nineteen papers (17 case reports and 2 case series) were included, describing 27 patients, 74.1% females, aged 18 to 69 years. COVID-19 was diagnosed by nasopharyngeal swab in 66.7% cases and required hospitalization in 11.1%. In 83.3% cases, SAT occurred after COVID-19. Neck pain was present in 92.6% cases and fever in 74.1%. Median TSH, fT3, and fT4 were 0.01 mU/l, 10.79 pmol/l, and 27.2 pmol/l, respectively. C-reactive-protein and erythrocyte sedimentation rate were elevated in 96% of cases. Typical ultrasonographic characteristics of SAT were observed in 83.3% of cases. Steroids were the most frequent SAT therapy. Complete remission of SAT was recorded in most cases. In conclusion, the size and quality of published data of SAT in COVID-19 patients are poor, with only case reports and case series being available. SAT clinical presentation in COVID-19 patients seems to be similar to what is generally expected.

## Introduction

Subacute thyroiditis (SAT) is a self-limited thyroid disease of viral or post-viral origin. SAT, also known as de Quervain thyroiditis, is typically characterized by a triphasic clinical course of thyrotoxicosis, hypothyroidism, and return to normal thyroid function. From a clinical point of view, SAT presents with neck pain typically with radiation to the ears and a wide spectrum of systemic symptoms, which include fever, asthenia, and malaise. In the initial phase, many patients also present clinical and/or biochemical manifestation of mild-moderate thyrotoxicosis, such as tremor and palpitations (1). Thyroid follicles are infiltrated, resulting in disrupted basement membrane and rupture of the follicles. This injury is thought to be the result of cytolytic T-cell recognition of viral and cell antigens (2).

While persistent hypothyroidism is a rare event, high circulating levels of inflammatory markers, such as C-reactive protein (CRP), and, more specifically, erythrocyte sedimentation rate (ESR) represent the most frequent biochemical finding at presentation (3, 4).

Several respiratory viruses, including coxsackievirus (5), mumps (6), Epstein–Barr virus (7), cytomegalovirus (8), and influenza virus (9), were reported to be associated with SAT development (10). However, owing to the fact that specific antiviral treatment is not required in most cases, diagnostic evaluations aimed at identifying the etiological viruses are not routinely performed in these patients. Thus, the viral or post-viral origin of SAT is supported by both direct and indirect evidences. Epidemiological data showed an overlap of seasonal outbreaks of infectious diseases and SAT outbreaks (6, 11). Indeed, high titers of virus-specific antibodies or positive virus swabs were found in patients harboring SAT and an association between presence of antibodies to specific viruses and SAT was observed (5, 6, 12). On the other hand, virus culture from thyroid tissue as well as viral RNA identification from thyroid cytological samples yielded conflicting results (13–15). At present, it is unclear whether follicle damage in SAT is caused by direct viral infection of the gland or by the host’s immunological response to the viral infection.

With the beginning of the coronavirus disease 2019 (COVID-19) pandemic caused by the severe acute respiratory syndrome coronavirus 2 (SARS-CoV-2), a thyroid impact was considered due to the potential of SARS-CoV-2 to cause multiorgan effects. Among the thyroid SARS-CoV-2 complications, SAT was early reported by some case report articles (16), and the question whether SAT might be an underestimated SARS-CoV-2 manifestation was raised (17). Subsequently, several original articles, editorials, and reviews were published on the thyroid sequelae experienced by patients with COVID-19 (18–22). More recently, a systematic review found that COVID-19 patients can develop thyroid dysfunction, frequently non-thyroidal illness syndrome, when hospitalized in an intensive care unit. Furthermore, several data supported the notion that having a thyroid disease would not increase the risk for SARS-CoV-2 infection, and thyroid patients do not need a COVID-19-adapted follow-up (23). According to the available data summarized in review articles, whether SAT can represent a COVID-19 complication remains an open question. Specifically, the prevalence of COVID-19-related SAT and the similarity of its clinical presentation to usual SAT remain unknown.

Therefore, the present study was conceived to summarize the published data about the association between COVID-19 and SAT. The literature was systematically reviewed to retrieve the largest number of original papers, case reports, and case series articles reporting SAT in patients diagnosed with SARS-CoV-2. The aims of the present study were to 1) explore the size and quality of the literature about SAT in COVID-19 and 2) evaluate the clinical characteristics of SAT in these patients.

## Materials and Methods

### Review Conduction

The systematic review was conducted according to the PRISMA statement (24), and the checklist is reported as supplemental file (**Supplemental File 1**).

### Search Strategy

A comprehensive computer literature search of the PubMed/MEDLINE, Embase, and Scopus databases was conducted to find published articles on the topic of our review. The search algorithm was created based on combinations of specific terms: (“De Quervain thyroiditis” OR “subacute thyroiditis”) AND (“SARS-CoV-2” OR COVID OR COVID-19 OR coronavirus). A beginning date limit was not used, and the search was updated until April 20, 2021, without language restrictions. To identify additional studies and expand our search, the references of the retrieved articles were also screened.

The authors declare that the study selection was conducted in the absence of any commercial or financial relationships that could be construed as a potential conflict of interest.

### Study Selection

**Figure 1:**
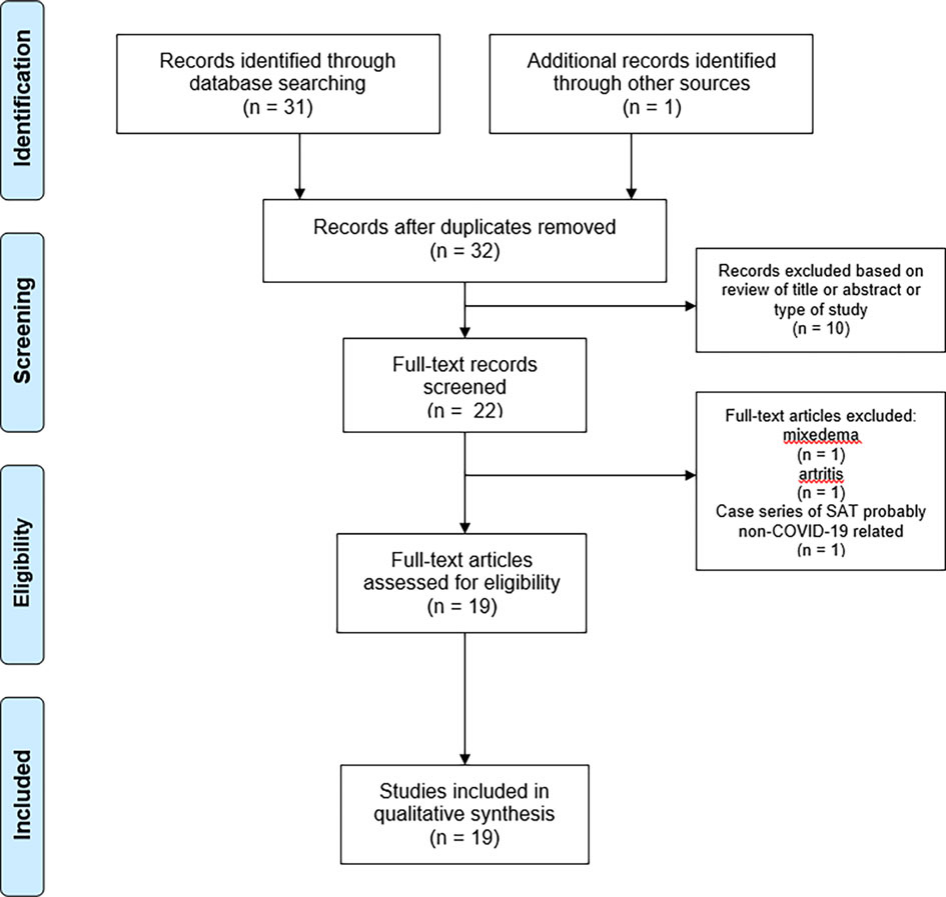
Search strategy and flow of articles.

Studies or subsets of studies that report data on the detection of SAT in patients with previous or concurrent occurrence of COVID-19 were eligible for inclusion. The main exclusion criteria were a) articles not within the field of interest of this review; b) review articles, editorials, or comments; c) articles that did not provide clear study characteristics or reports that had overlapping patient data; and d) cases in which the SAT diagnosis was not clearly rendered. Two authors (LC, LS) independently reviewed the titles and abstracts of the retrieved articles, applying the inclusion and abovementioned exclusion criteria. Then, the same two researchers independently reviewed the full text of the articles to determine their final inclusion. Disagreements were solved in a final mutual meeting involving also other two authors (PT, MR). **Figure 1** illustrates the research strategy and flow of articles.

### Data Extraction

For each included study, information was extracted concerning reference data (authors, journal, year of publication, and country of origin). Number of patients evaluated, clinical data regarding COVID-19 presentation and SAT presentation, biochemical evaluation of thyroid function parameters, therapy for subacute thyroiditis, and long-term follow-up data were also extracted.

Each case report and case series were carefully evaluated to verify that no patient was included in more than one study.

## Results

### Retrieved Articles

Using the above search strategy, 32 records were initially found. Among these, 14 were excluded because they did not fit with the study aim while the remaining 18 (16, 17, 25–41) were included in the systematic review. Another paper (40), not included in the initial pool of retrieved records, was added to the systematic review because it was known by the authors. Finally, 19 articles, consisting of 17 case reports and 2 case series (with four and six SAT cases), were included. Remarkably, no original articles including large sample size were found. **Figure 1** illustrates the flow of articles.

### General Features of Included Articles

The 19 articles included in the systematic review included a total number of 27 SAT in patients diagnosed with COVID-19. The 19 studies were published from May 21, 2020 (16), to April 14, 2021 (41), by authors from 10 countries: seven cases from Italy, seven from Iran, four from the United States of America, two from Spain, two form India, one from Mexico, one from the Philippines, one from Singapore, one from Turkey, and one from the United Kingdom. The main demographic and clinical characteristics (regarding COVID-19 and SAT presentation) of each included patient are summarized in **Table 1**.

**Table 1:** Description of the anagraphic characteristics and clinical presentation of COVID-19 and SAT of the 27 included patients.

Year	N.	Reference	Country	Age	Gender	Preexisting Thyroid Disease	COVID-19 Diagnosis	Clinical presentation of COVID-19	Onset after COVID-19 (days)	General	Neck pain	Fever	Palpitations	Therapy
2020	1	(32)	Spain	46	F	NO	Positive IgG	Asymptomatic		YES	YES	YES	NO	Prednisone (40 mg/day as the starting dose, gradually tapered)
2020	2	(25)	Turkey	41	F	NO	RT-PCR	Asymptomatic	0	NO	YES	YES	NO	Prednisolone 16 mg daily
2020	3	(16)	Italy	18	F	NO	RT-PCR	Mild upper respiratory symptoms (rhinorrhea and cough)	19	YES	YES	YES	YES	Prednisone (25 mg/day as the starting dose, gradually tapered)
2020	4	(17)	Italy	38	F	NO	RT-PCR	Mild upper respiratory symptoms	16	YES	YES	YES	YES*^c^*	Prednisone (25 mg/day as the starting dose, gradually tapered)
2020	5	(17)	Italy	29	F	NO	Positive IgG	Rhinorrhea	30	YES	YES	YES	YES	Prednisone (25 mg/day as the starting dose, gradually tapered), propranolol 40 mg/day
2020	6	(17)	Italy	29	F	NO	RT-PCR	Fever, cough, rhinorrhea, anosmia	36	YES	YES	NO	YES	Ibuprofen 600 mg/day
2020	7	(17)	Italy	46	F	NO	RT-PR	Fever, cough, rhinorrhea, anosmia, asthenia	20	YES	YES	YES	YES	Prednisone (25 mg/day as the starting dose, gradually tapered)
2020	8	(28)	Mexico	37	F	NO	RT-PCR	Odynophagia and anosmia	30	YES	YES	NO	NO	No treatment (still thyrotoxic after 1 month)
2020	9	(39)	India	58	M	NO	RT-PCR	Fever	0	NO	YES	YES	YES	Prednisolone (30 mg/day as the starting dose, gradually tapered), propranolol 40 mg/day
2021	10	(29)	USA	37	M	NO	RT-PCR	Productive cough, fever, chills, dyspnea	30	YES	YES	NO	YES	Aspirin, propranolol
2021	11	(37)	Iran	33	M	NO	RT-PCR	Interstitial pneumonia	8	YES	YES	YES	YES	Dexamethasone 4 mg every 8 h for 5 days, then oral prednisone 25 mg daily with tapering
2021	12	(34)	UK	57	F	NO	Likely diagnosis*^a^*	Mild upper respiratory symptoms, anosmia	60	YES	YES	NO	YES	Ibuprofen 200 mg three times per day and paracetamol 1 g three times per day
2020	13	(38)	Italy	69	F	NO	RT-PCR	Interstitial pneumonia	5	YES	NO*^b^*	NO	YES	First methimazole, then shifted to methylprednisolone 40 mg/die for 3 days, then prednisone 25 mg/die progressively tapered
2021	14	(36)	USA	41	F	NO	RT-PCR	Fever, cough, and coryza	14	YES	YES	YES	YES	Ibuprofen 600 mg every 6 h and prednisone 40 mg daily.
2021	15	(41)	USA	67	M	NO	RT-PCR	Heart failure and interstitial pneumonia	0	YES	NO	YES	YES	Methimazole for 1 month, after no recovery shifted to prednisone
2020	16	(27)	Singapore	34	M	NO	RT-PCR	Fever, mild upper respiratory symptoms, anosmia	3	NO	YES	NO	YES	Prednisolone (20 mg/day as starting dose, gradually tapered)
2020	17	(33)	USA	29	F	NO	RT-PCR	Mild upper respiratory symptoms	49	YES	YES	YES	YES	Prednisone (40 mg/day as the starting dose, gradually tapered), atenolol 50 mg/die
2020	18	(30)	Spain	28	F	NO	RT-PCR	Diarrhea, abdominal pain	14	YES	YES	YES	YES	Aspirin 500 mg and propranolol 40 mg every 6 hours
2020	19	(26)	Italy	43	F	NO	RT-PCR	Fever, mild upper respiratory symptoms	45	YES	YES	YES	YES	Prednisone (25 mg/day as the starting dose, gradually tapered)
2021	20	(40)	India	29	F	NO	RT-PCR	Fever, cough, and other flu-like symptoms	45	NO	YES	YES	YES	Indomethacin 25 mg and propanol 40 mg thrice daily
2020	21	(31)	Philippines	47	F	NO	RT-PCR	Lobar pneumonia	-47	YES	YES	NO	NO	Mefenamic acid, later shifted to celecoxib
2021	22	(35)	Iran	37	F	NO	Positive IgG	Myalgia for a few days	30	YES	YES	YES	YES	Prednisone (25 mg/day as the starting dose, gradually tapered)
2021	23	(35)	Iran	35	M	NO	Positive IgG	Asymptomatic		YES	YES	YES	YES	Prednisone (25 mg/day as the starting dose, gradually tapered)
2021	24	(35)	Iran	41	F	NO	Positive IgG	Low-grade fever and mild myalgia for few days	30	YES	YES	YES	YES	Prednisone (25 mg/day as the starting dose, gradually tapered)
2021	25	(35)	Iran	52	M	NO	Positive IgG	Low-grade fever, dry cough, and mild myalgia for few days	30	YES	YES	YES	YES	Prednisone (25 mg/day as the starting dose, gradually tapered)
2021	26	(35)	Iran	34	F	NO	Positive IgG	Asymptomatic		YES	YES	YES	YES	Prednisone (25 mg/day as the starting dose, gradually tapered)
2021	27	(35)	Iran	26	F	NO	Positive IgG	Self-limited dry cough for 1 week	30	YES	YES	YES	YES	Prednisone (25 mg/day as the starting dose, gradually tapered)

RT-PCR, real-time polymerase chain reaction; COVID-19, coronavirus disease 2019; SAT, subacute thyroiditis.

^a^No direct demonstration of SARS-CoV-2.

^b^While on morphine for back surgery.

^c^Atrial fibrillation.

### Demographic Features of Patients

Twenty (74.1%) patients were females and seven (25.9%) males. Patients’ age at SAT occurrence ranged from 18 to 69 years (median 37.5, IQR 33–46). No patient was previously diagnosed with thyroid disease.

### COVID-19 Diagnosis and Presentation

COVID-19 diagnosis was rendered through real-time polymerase chain reaction (RT-PCR) on nasopharyngeal swab in 18 cases (66.7%), and by positivity of specific IgG in eight cases (29.6%). In one case, COVID-19 was suspected based on the presence of its typical symptoms (anosmia, dysgeusia) with no direct evidence of SARS-CoV-2 infection. COVID-19 was asymptomatic in 3 cases (11.1%), with mild upper respiratory symptoms in 21 cases (77.7%), and with pneumonia requiring hospitalization in 3 cases (11.1%). SARS-CoV-2-related peculiar symptoms (i.e., anosmia or dysgeusia) were reported in 5 (18.5%) out of 27 cases.

### Time Interval Between SAT Occurrence and COVID-19 Diagnosis

The timing of SAT diagnosis with respect to COVID-19 was described in 24 cases:

In 20 (83.3%) cases, SAT occurred after COVID-19 onset, after a median of 30 (IQR 16–32) days. In three (12.5%) cases, SAT and COVID-19 were synchronous. In one (4.2%) patient (N # 21), the onset of SAT preceded by 47 days the molecular confirmation of COVID-19. This latter case should be briefly overviewed. At SAT diagnosis, the patient did not have fever or respiratory symptoms but a right lower lobe pneumonia was found on a chest radiography. Unfortunately, the reverse transcription-polymerase chain reaction for SARS-CoV-2 using nasopharyngeal and oropharyngeal swabs was performed only after several weeks after the onset of SAT symptoms and found positive. Although the possibility that SARS-CoV-2 infection was already present at the onset of SAT cannot be ascertained, the authors suggested that SAT might occur also in patients with confirmed COVID-19 without respiratory manifestations.

### SAT Clinical Presentation

Neck pain was present in 25 (92.6%) cases; notably, one of the two patients without neck pain was taking opioid drugs since a recent surgical intervention (N. #13). General symptomatology (i.e., asthenia and malaise) was described in 23 (85.2%) patients. Fever was recorded in 20 (74.1%) patients. Palpitations were experienced by 22 (81.5%) patients, and one presented a new-onset episode of atrial fibrillation.

### Thyroid Laboratory Tests and Inflammation Markers

Serum assessment was quite heterogeneous among the 19 studies. Available data are summarized in **Table 2**.

**Table 2:** Biochemical evaluation of thyroid function parameters, thyroglobulin, thyroid autoantibodies, and inflammatory markers in the 27 included patients.

Year	N.	Ref	TSH	TSH ref range	FT3	FT3 ref range	FT4	FT4 ref range	Tg	TgAb	TPOAb	TRAb	CRP/ESR
2020	1	(32)	0.11	0.5–4.78 mIU/ml	N/A	N/A	2.18	0.89–1.76 ng/dl	N/A	N/A	Slightly positive	N/A	High
2020	2	(25)	<0.008	N/A	7.7	3.1–6.8 pmol/l	25.7	12–21 pmol/l	N/A	0	0	0	High
2020	3	(16)	<0.04	0.5–4.1 mIU/l	8.7	4.6–8.4 pmol/l	27.2	11–23 nmol/l	5.6	120 UI/ml (positive)	0	0	High
2020	4	(17)	0.1	0.4–4.5 mIU/ml	8.0	2.3 to 4.2 pmol/l	29.3	6–16 pmol/l	75.3	0	0	0	High
2020	5	(17)	< 0.01	0.4–4.5 mIU/ml	8.9	2.3 to 4.2 pmol/l	31.8	6–16 pmol/l	80	38 (positive)	0	0	High
2020	6	(17)	N/A	N/A	N/A	N/A	N/A	N/A	N/A	N/A	N/A	N/A	N/A
2020	7	(17)	<0.01	0.4–4.5 mIU/ml	6.9	2.3 to 4.2 pmol/l	27.8	6–16 pmol/l	N/A	N/A	N/A	0	Normal
2020	8	(28)	<0.001	N/A	211^a^	80–150 ng/dl^a^	1.6	0.7–1.48 ng/dl	N/A	0	0	N/A	High
2020	9	(39)	<0.005	0.27–4.2 mIU/l	2.88^a^	0.80–2.0 ng/ml^a^	20.11^b^	5.10–14.1 μg/dl^b^	N/A	N/A	N/A	N/A	High
2021	10	(29)	0.01	0.4–4.5 μIU/ml	202^a^	80–150 ng/dl^a^	2.3	0.6–1.3 ng/dl	N/A	N/A	0	0	High
2021	11	(37)	<0.001	N/A	236 ^a^	75–195 ng/dl ^a^	23.1^b^	4–11 μg/dl^b^	N/A	N/A	0	0	High
2021	12	(34)	0.1	0.27–4.2 mU/l	N/A	N/A	21.2	12–22 pmol/l	N/A	6.61 U/ml (positive)	71.8 UI/ml (positive)	0	High
2020	13	(38)	0.08	0.27–4.2 mU/l	5.5	2–4.4 pg/ml	24.6	0.3–17 pg/ml	187	N/A	N/A	N/A	N/A
2021	14	(36)	<0.008	0.7–4.20 mIU/l	3.39^a^	1.232–3.08 nmol/l^a^	60.63	11.61–23.22 pmol/l	N/A	N/A	96.71 (positive)	0	High
2021	15	(41)	0.029	0.27–4.2 μIU/ml	1.2^a^	0.80–2.0 ng/ml^a^	2.1	0.8–1.7 ng/dl	N/A	0	0	0	High
2020	16	(27)	<0.01	0.65–3.70 mU/l	13.4	3.2–5.3 pmol/l	41.8	8.8–14.4 pmol/l	N/A	N/A	0	0	High
2020	17	(33)	0.01	mU/l	374^a^	80–150 ng/l^a^	4.4	0.6–1.3 ng/l	N/A	N/A	0	0	High
2020	18	(30)	<0.001	0.38–5.33 mU/l	N/A	N/A	37.5	7.0–16.0 pmol/l	N/A	0	0	0	High
2020	19	(26)	0.006	0.27–4.2 mU/l	7.03	1.71–3.71 pg/ml	2.69	0.7–1.48 ng/dl	188	0	0	0	High
2021	20	(40)	0.007	N/A	5.05^a^	ng/ml^a^	7.77	ng/dl	N/A	0	0	0	High
2020	21	(31)	0.05	0.47–4.68 μIU/ml	1.4^a^	0.97–1.69 ng/ml ^a^	1.68	0.78–2.19 pg/ml	N/A	0	0	0	High
2021	22	(35)	<0.01	0.4–4.0 mU/l	25.4	3.1–6.8 pmol/l	2.3	12–21 pmol/l	N/A	N/A	N/A	N/A	High
2021	23	(35)	0.12	0.4–4.0 mU/l	19.3	3.1–6.8 pmol/l	24.7	12–21 pmol/l	N/A	N/A	N/A	N/A	High
2021	24	(35)	<0.01	0.4–4.0 mU/l	23.7	3.1–6.8 pmol/l	21.9	12–21 pmol/l	N/A	N/A	N/A	N/A	High
2021	25	(35)	0.17	0.4–4.0 mU/l	21.6	3.1–6.8 pmol/l	26.7	12–21 pmol/l	N/A	N/A	N/A	N/A	High
2021	26	(35)	0.23	0.4–4.0 mU/l	18.1	3.1–6.8 pmol/l	18.4	12–21 pmol/l	N/A	N/A	N/A	N/A	High
2021	27	(35)	0.07	0.4–4.0 mU/l	18.9	3.1–6.8 pmol/l	19.5	12–21 pmol/l	N/A	N/A	N/A	N/A	High

N/A, not available; TgAb, anti-thyroglobulin antibodies; TPOAb, anti-thyroperoxydase antibodies; TRAb, anti-TSH-receptor antibodies; CRP/ESR, C-reactive protein/erythrocyte sedimentation rate.

^a^Total T3.

^b^Total T4.

Thyroid function tests were performed in all patients but one and showed overt thyrotoxicosis in all cases. Particularly, median TSH (available in 26 out of 27 cases) was 0.01 mU/l (IQR 0.008–0.07), median free-T4 (23 cases) was 27.2 pmol/l (IQR 22.4-31.1) with a median increase of 1.27 times (IQR 1.17–1.83) the upper limit of the reference range, and median-free T3 (15 cases) was 10.79 pmol/l (IQR 8.5–19.1) with a median increase of 2.11 times (IQR 1.67–2.80) of the upper limit of the reference range. Total T4 was measured in three (11.1%) patients ranging from 13.5 to 23.1 μg/dl. Total T3 was measured in eight (29.6%) patients with a median value of 2.15 ng/ml (IQR 1.865–2.49).

Thyroid autoantibodies were measured in 18 (66.6%) of cases. Indeed, anti-thyroglobulin antibodies (TgAb) and anti-thyroperoxydase antibodies (TPOAb) can be detected in some non-autoimmune thyroid diseases, such as SAT (42). Although some studies reported the *de novo* appearance of TgAb in up to 25%–50% of SAT patients, and, to a lesser extent, TPOAb, this positivity is usually transient and most patients do not develop autoimmune sequelae (43). Moreover, anti TSH-receptor antibody (TRAb) testing is performed in some patients in the thyrotoxic phase of SAT to exclude the presence of Graves’ disease.

Positive TgAb tests were found in 3 out of 11 patients in whom they were measured. Positive TPOAb tests were found in 3 out of 16 patients. TRAb measurement was performed in 15 patients with negative results in all cases. Thyroglobulin was measured in five (18.5%) patients and was always elevated.

SAT-specific inflammation markers, such as CRP and ESR, were performed in 25 patients and were high in 24 (96%).

### Thyroid-Specific Imaging

The results of thyroid-specific imaging performed for the included patients are summarized in **Table 3**. Thyroid ultrasound was performed in 24 cases (88.9%). The typical ultrasonographic characteristics of SAT (patchy hypoechogenic areas with reduced vascularization) were observed in 20 cases, while in the remaining cases non-specific patterns were described. Thyroid ^99m^Tc-pertechnetate scintigraphy was performed in nine (33.3%) patients, with the uptake being either reduced (four cases) or absent (five cases).

**Table 3:** Results of thyroid-specific imaging in the 27 included patients.

Year	N.	Ref	Ultrasound	US typical for SAT	Scintigraphy
2020	1	(32)	Enlarged thyroid with heterogeneous echotexture	NO	Reduced uptake
2020	2	(25)	Increased vascularity, heterogeneous parenchyma	NO	N/A
2020	3	(16)	Multiple, diffuse hypoechoic areas	YES	N/A
2020	4	(17)	Enlarged thyroid gland with multiple hypoechoic areas and absent vascularization at color Doppler	YES	N/A
2020	5	(17)	Increased thyroid volume with bilateral diffuse hypoechoic areas and absent vascularization at color Doppler ultrasonography	YES	No uptake
2020	6	(17)	Increased thyroid volume (25 ml) with bilateral diffuse hypoechoic areas	YES	N/A
2020	7	(17)	Increased thyroid volume (18 ml) with bilateral diffuse hypoechoic areas and absent to mild vascularization at color Doppler ultrasonography	YES	N/A
2020	8	(28)	N/A		No uptake
2020	9	(39)	Diffuse bilateral enlargement of thyroid with hypoechogenicity and increased vascularity on color Doppler	YES	Reduced uptake
2021	10	(29)	Diffusely heterogeneous echotexture	NO	N/A
2021	11	(37)	Bilateral ill-defined hypoechoic areas	YES	N/A
2021	12	(34)	Patchy areas of variably-reduced parenchymal echogenicity bilaterally	YES	Reduced uptake
2020	13	(38)	Enlarged hypoechoic thyroid, decreased vascularity	YES	No uptake
2021	14	(36)	Heterogeneous thyroid gland with bilateral patchy ill-defined hypoechoic areas	YES	N/A
2021	15	(41)	Mildly enlarged thyroid gland with no increased vascularity and 5-mm bilateral cysts	NO	N/A
2020	16	(27)	Enlarged thyroid gland with heterogeneous echotexture; hypoechoic areas with ill-defined margins corresponding to the hard regions palpable. Reduced blood flow in both lobes	YES	N/A
2020	17	(33)	N/A		N/A
2020	18	(30)	N/A		No uptake
2020	19	(26)	Diffusely enlarged and hypoechogenic thyroid gland.	YES	Reduced uptake
2021	20	(40)	Enlarged heterogeneously hypoechoic left lobe of thyroid and isthmus with normal vascularity, and bulky right lobe of thyroid with few ill-defined hypoechoic areas which suggestive of thyroiditis.	YES	No uptake
2020	21	(31)	Slightly enlarged right thyroid lobe, with ill-defined hypoechogenicity and normal vascularity in both lobes	YES	N/A
2021	22	(35)	Hypoechoic areas	YES	N/A
2021	23	(35)	Hypoechoic areas	YES	N/A
2021	24	(35)	Hypoechoic areas	YES	N/A
2021	25	(35)	Hypoechoic areas	YES	N/A
2021	26	(35)	Hypoechoic areas	YES	N/A
2021	27	(35)	Hypoechoic areas	YES	N/A

N/A, not available; US, ultrasound.

### SAT Treatment

Steroidal therapy was the most frequently administered therapy for SAT: the most used agent was prednisone (in 13 cases), but less frequently dexamethasone (in one case), prednisolone (in two cases), and methylprednisolone (in one case) were used. The median of prednisone equivalents administered per day was 25 mg (IQR 25–35). Seven patients received non-steroidal anti-inflammatory agents (including aspirin, indomethacin, and mefenamic acid). These therapies were often paired with beta-blockers (most frequently propranolol). Two patients with thyrotoxicosis were initially treated with anti-thyroid drugs and subsequently switched to steroid therapy.

### SAT Outcome

A complete resolution of symptoms and thyrotoxicosis was recorded in most cases, with the exception of four patients (14.8%) in whom an evolution toward subclinical hypothyroidism was witnessed and another one who developed overt hypothyroidism.

### Risk of Bias

Data extracted from the articles were almost complete in all the above outcomes except that regarding the laboratory tests. In fact, unfortunately, the antibody profile was reported in less than 50% of cases with a significant risk of bias due to missing results. On the contrary, the risk of bias was negligible in all the other outcomes.

### Summary of Findings

The synthesis of results was made according to PRISMA statement (24). As reported in **Table 4**, based on the data found in the literature, the certainty of evidence was moderate in all outcomes except that of laboratory tests, which was lower.

**Table 4:** Summary of findings about COVID-19-associated SAT.

Outcome	Participants (n)	Certainty of the evidence	Comments
Gender	27	Moderate	SAT more often recurs in women
Presentation of COVID-19	27	Moderate	In patients with SAT COVID-19 usually presents with mild upper respiratory symptoms
Time interval between SAT and COVID-19	24	Moderate	SAT typically manifests 3–60 days after COVID-19
SAT clinical presentation	27	Moderate	COVID-19-associated SAT usually manifests as classical SAT (i.e., neck pain, asthenia and malaise, palpitations, fever)
Thyroid laboratory tests and inflammation markers	Variable	Low to moderate	Overt thyrotoxicosis is present in all cases of SAT; CRP and ESR, and thyroglobulin are typically elevated
Thyroid US	24	Moderate	The ultrasonographic characteristics of classical SAT (patchy hypoechogenic areas with reduced vascularization) are observed in COVID-19-associated SAT
SAT treatment	27	Moderate	Steroidal therapy was the most frequently administered therapy
SAT outcome	27	Moderate	A complete resolution of symptoms and euthyroidism is reached in most cases

SAT, subacute thyroiditis; US, ultrasound; CRP, C-reactive protein; ESR, erythrocyte sedimentation rate.

GRADE Working Group grades of evidence.

High certainty: we are very confident that the true effect lies close to that of the estimate of the effect.

Moderate certainty: we are moderately confident in the effect estimate: The true effect is likely to be close to the estimate of the effect, but there is a possibility that it is substantially different.

Low certainty: our confidence in the effect estimate is limited; the true effect may be substantially different from the estimate of the effect.

Very low certainty: we have very little confidence in the effect estimate. The true effect is likely to be substantially different from the estimate of effect.

## Discussion

SAT is generally secondary to upper respiratory tract infections by several viruses (3, 10); thus, SAT might represent a potential complication of SARS-CoV-2 infection. The fact that SARS-CoV-2 recognizes angiotensin-converting enzyme 2 (ACE-2) as its cellular entry receptor (44) and the recent demonstration of ACE-2 expression in follicular thyroid cells would further support this possibility (45–47).

Here we conceived a systematic review to achieve more solid evidence about the relationship between SAT and COVID-19. Particularly, we aimed to evaluate the size and quality of the published literature on this topic, and the clinical characteristics of SAT in this setting of patients.

Regarding the first objective, we found 19 studies reporting a total of 27 patients with SAT. Surprisingly, we did not find original articles with large sample size although the first case report was published 1 year ago (16). The 17 case reports (16, 25–34, 36–41) and the 2 case series (17, 35) described carefully the history of patients and their SAT. In this context, it should be highlighted that two recent systematic reviews on a similar topic included 21 (48) and 17 (49) SAT cases, respectively. Our systematic review, conducted 3 months later and conceived with two specific aims, found 27 cases. The fact that the number of reported SAT cases in literature did not exponentially increase in a time frame during which COVID-19 cases significantly increased worldwide indirectly confirms that SAT is a rare complication of COVID-19. Furthermore, because of the lack of large-sample studies, both size and quality of the literature about SAT in COVID-19 patients have to be considered poor. From this point of view, taking up the pertinent question raised by Brancatella et al. (17) of whether SAT is an underestimated manifestation of SARS-CoV-2, the issue remains unsolved. A most reasonable answer would be that SAT represents, at best, a rare complication of COVID-19.

Considering the second aim of the present systematic review, SAT occurred generally after COVID-19. Its clinical presentation appears to be similar to “classic” forms of SAT, encompassing neck pain, asthenia/malaise, fever, and palpitations. Under a biochemical point of view, the reported patients always presented with thyrotoxicosis, usually with elevated serum inflammation markers. When available, the ultrasonographic/scintigraphic features of these patients were generally typical of virus-related thyroiditis. In addition, most patients were treated with steroids with complete resolution of symptoms. It should be noted that the median initial dose of prednisone employed in the reported patients (25 mg/day) is lower than the one recommended by the most recent guidelines (40 mg daily) (50). In general, patients with post-COVID-19 SAT presented a moderate-mild form of the disease, in terms of both clinical and biochemical presentation, with no peculiar clinical features.

The COVID-19 presentation and severity in the 27 patients with SAT deserve to be discussed. First, some specific and peculiar symptoms of COVID-19, such as anosmia and/or dysgeusia, were reported in a minority of SAT patients (19%). It is worth underlining that the above symptoms received great informative emphasis, making it unlikely that they were left unrecognized. Second, most patients experienced a pauci- or asymptomatic COVID-19 disease, with only three patients requiring hospitalization, and that their median age was 37 years, much younger than the typical COVID-19 hospitalized patients. Moreover, the female-to-male ratio was high, this being the opposite of what happens in a case series of hospitalized COVID-19 patients (51). In this context, it should be highlighted that in a recent study by Trimboli et al. (52) aimed at searching for an association between SAT and SARS-CoV-2 in COVID-19-specific symptoms and contact tracing data, it was found that among the 10 included SAT patients, none had positive SARS-CoV-2 diagnostic tests, and only one case had a contact with people who were diagnosed with SARS-CoV-2.

To date, several large series studies as well as several reviews have evaluated the thyroid impact in COVID-19 hospitalized/discharged patients (18–22). Surprisingly, even if these large case series (18–20, 23) were specifically aimed at describing the thyroidal repercussions of COVID-19, no occurrence of SAT was reported, allowing the following speculation. It could be that, among the hospitalized COVID-19 patients, the typical symptoms of SAT were misdiagnosed in severe COVID-19 disease or masked by the routine use of high-dose corticosteroids currently employed in critical COVID-19 patients (53). This hypothesis would hold particularly true only for the so-called “second wave” of the pandemic, since corticosteroids were contraindicated by most national guidelines in the early phase of the pandemic (54). It could be thus hypothesized that SAT would be a typical complication of less severe forms of COVID-19 occurring in community-dwelling, young female subjects that are less frequently included in clinical studies, while hospitalized patients would more often experience non-thyroidal illness syndrome due to the hyperinflammatory state typical of severe COVID-19 (55–57). In this context, a systematic review by Ruggeri et al. (58) provided a comprehensive review of all COVID-19-related inflammatory disorders. This paper highlighted, from a slightly different point of view, that thyroid dysfunction is frequently observed in COVID-19 patients, regardless of the underlying thyroid disease, and physicians should be aware of its possible occurrence. The fact that no extreme increases in the number of SAT cases occurred in the last months, even in areas greatly affected by COVID-19 (59), could be due to the fact that social distancing measures and mask use reduced the diffusion of other SAT-causative viruses, similarly to what happened with the H1N1 virus (60).

The here reviewed evidence has several limitations: even if the clues in favor of a SARS-CoV-2-induced SAT exist, the size of published cases is poor, with only case reports and case series being available. Also, our review process was greatly limited by the nature of the available literature and by the lack of uniformity in part of the data, mainly the laboratory tests performed.

These results can give evidence-based information to help clinicians who encounter cases of COVID-19-related SAT in everyday practice. In particular, available literature suggests that these forms of SAT are rather mild and do not require any specific treatment when compared with “classic” SAT forms. Nevertheless, this complication does not appear to be particularly frequent in COVID-19 patients, especially in those who require hospitalization. More data regarding larger series of patients with a more uniform evaluation of thyroid function parameters will be required to draw more firm conclusions on the real incidence of SAT in COVID-19 patients. Moreover, long-term follow-up data will indicate if patients who experience COVID-19-related SAT are at risk for long-term thyroid sequelae.

In conclusion, the size of published data of SAT in COVID-19 patients is poor and the SAT clinical presentation in COVID-19 patients appears overall similar from that generally expected. According to these evidence-based data, SAT cannot be considered as a direct or frequent complication of SARS-CoV-2. However, since the rapid worldwide diffusion of SARS-CoV-2 and its variants, the present findings might change in the next future.

## Data Availability Statement

The original contributions presented in the study are included in the article/**Supplementary Material**. Further inquiries can be directed to the corresponding author.

## Author Contributions

PT, LCr, and MR designed and conceptualized the study, analyzed the data, and drafted the manuscript for intellectual content; all the co-authors interpreted the data and revised the manuscript for intellectual content. All authors contributed to the article and approved the submitted version.

## Conflict of Interest

The authors declare that the research was conducted in the absence of any commercial or financial relationships that could be construed as a potential conflict of interest.

## Publisher’s Note

All claims expressed in this article are solely those of the authors and do not necessarily represent those of their affiliated organizations, or those of the publisher, the editors and the reviewers. Any product that may be evaluated in this article, or claim that may be made by its manufacturer, is not guaranteed or endorsed by the publisher.

## Abbreviations

COVID-19, Coronavirus Disease 2019; CRP, C-reactive protein; ESR, erythrocyte sedimentation rate; RT-PCR, real-time polymerase chain reaction; SARS-CoV-2, severe acute respiratory syndrome coronavirus 2; SAT, subacute thyroiditis; T3, triiodothyronine; T4, thyroxine; TgAb, anti-thyroglobulin antibodies; TPOAb, anti-thyroperoxidase antibodies; TSH, thyroid-stimulating hormone.
